# Evolving DNA repair synthetic lethality targets in cancer

**DOI:** 10.1042/BSR20221713

**Published:** 2022-12-16

**Authors:** Sanat Kulkarni, Juliette Brownlie, Jennie N. Jeyapalan, Nigel P. Mongan, Emad A. Rakha, Srinivasan Madhusudan

**Affiliations:** 1 Department of Medicine, Sandwell and West Birmingham Hospitals, Lyndon, West Bromwich B71 4HJ, U.K.; 2 Academic Unit of Translational Medical Sciences, Nottingham Biodiscovery Institute, School of Medicine, University of Nottingham, University Park, Nottingham NG7 3RD, U.K.; 3 Department of Pathology, Nottingham University Hospitals, Nottingham NG51PB, U.K.; 4 Department of Oncology, Nottingham University Hospitals, Nottingham NG51PB, U.K.

**Keywords:** ATM inhibitors, ATR inhibitors, DNA synthesis and repair, PARP, PARP inhibitors, Synthetic lethality

## Abstract

DNA damage signaling response and repair (DDR) is a critical defense mechanism against genomic instability. Impaired DNA repair capacity is an important risk factor for cancer development. On the other hand, up-regulation of DDR mechanisms is a feature of cancer chemotherapy and radiotherapy resistance. Advances in our understanding of DDR and its complex role in cancer has led to several translational DNA repair-targeted investigations culminating in clinically viable precision oncology strategy using poly(ADP-ribose) polymerase (PARP) inhibitors in breast, ovarian, pancreatic, and prostate cancers. While PARP directed synthetic lethality has improved outcomes for many patients, the lack of sustained clinical response and the development of resistance pose significant clinical challenges. Therefore, the search for additional DDR-directed drug targets and novel synthetic lethality approaches is highly desirable and is an area of intense preclinical and clinical investigation. Here, we provide an overview of the mammalian DNA repair pathways and then focus on current state of PARP inhibitors (PARPi) and other emerging DNA repair inhibitors for synthetic lethality in cancer.

## Introduction

### DNA damage

**Figure 1:**
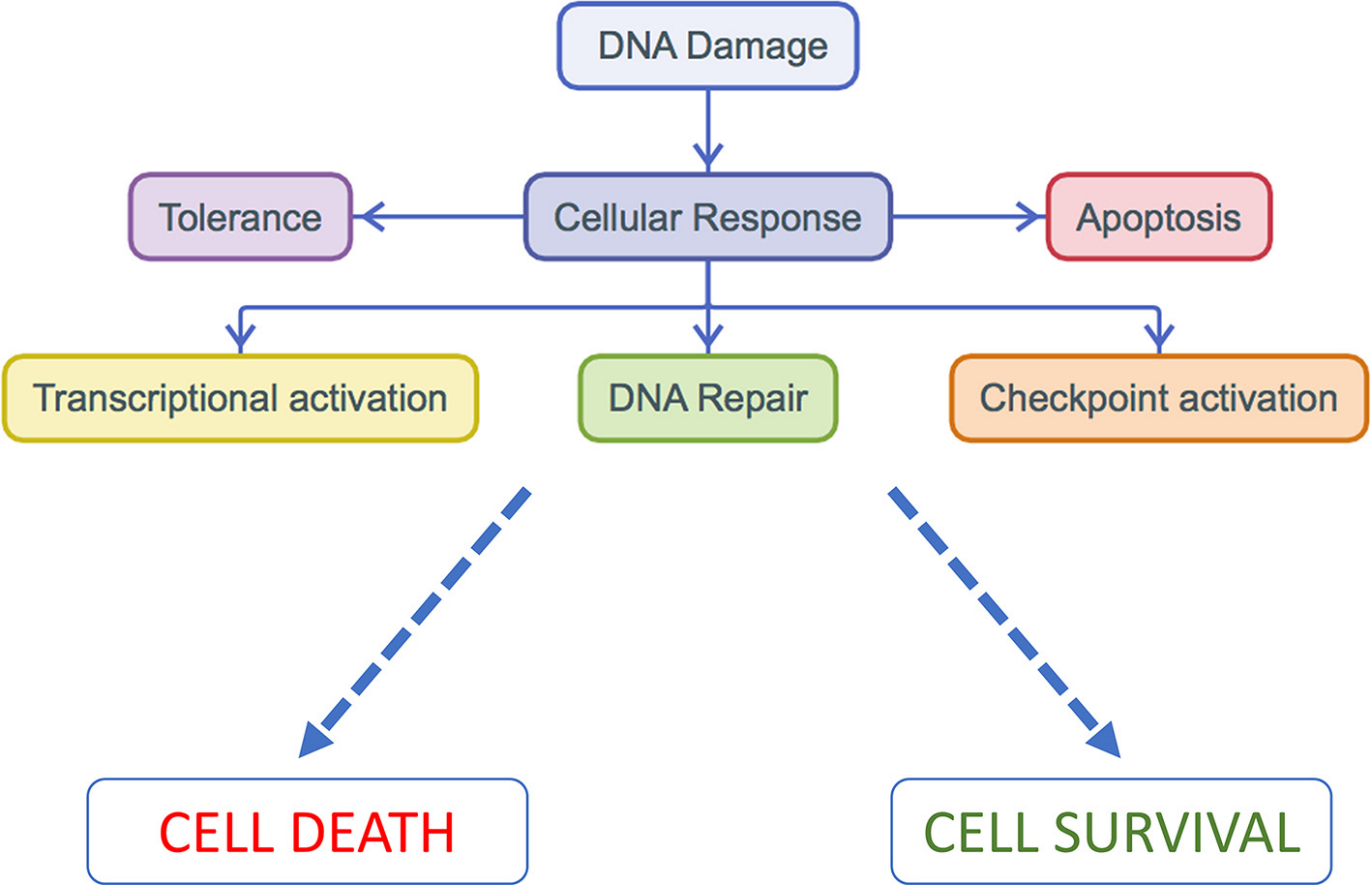
Cellular response to DNA damage

DNA repair pathways are essential for maintenance of genomic integrity, loss of which can promote carcinogenesis and influence response to cancer treatments. DNA damage occurs constantly due to endogenous and exogenous causes. Endogenous causes include reactive oxygen species (ROS), spontaneous base modifications, and errors during DNA replication. Exogenous causes include ultraviolet (UV) light, ionizing radiation, and chemicals, including chemotherapeutic agents. Therefore, in order to maintain genomic integrity, both prokaryotes and eukaryotes have evolved highly conserved DNA repair mechanisms to identify and correct DNA damage [1]. Following detection of DNA damage, cells may initiate different pathways, dependent on the type of damage, resulting in either: tolerance of the damage, transcriptional activation, induction of apoptosis (for highly damaged cells), or cell cycle arrest with subsequent repair of the DNA lesion [2,3] (Figure 1).

### DNA repair pathways

**Figure 2:**
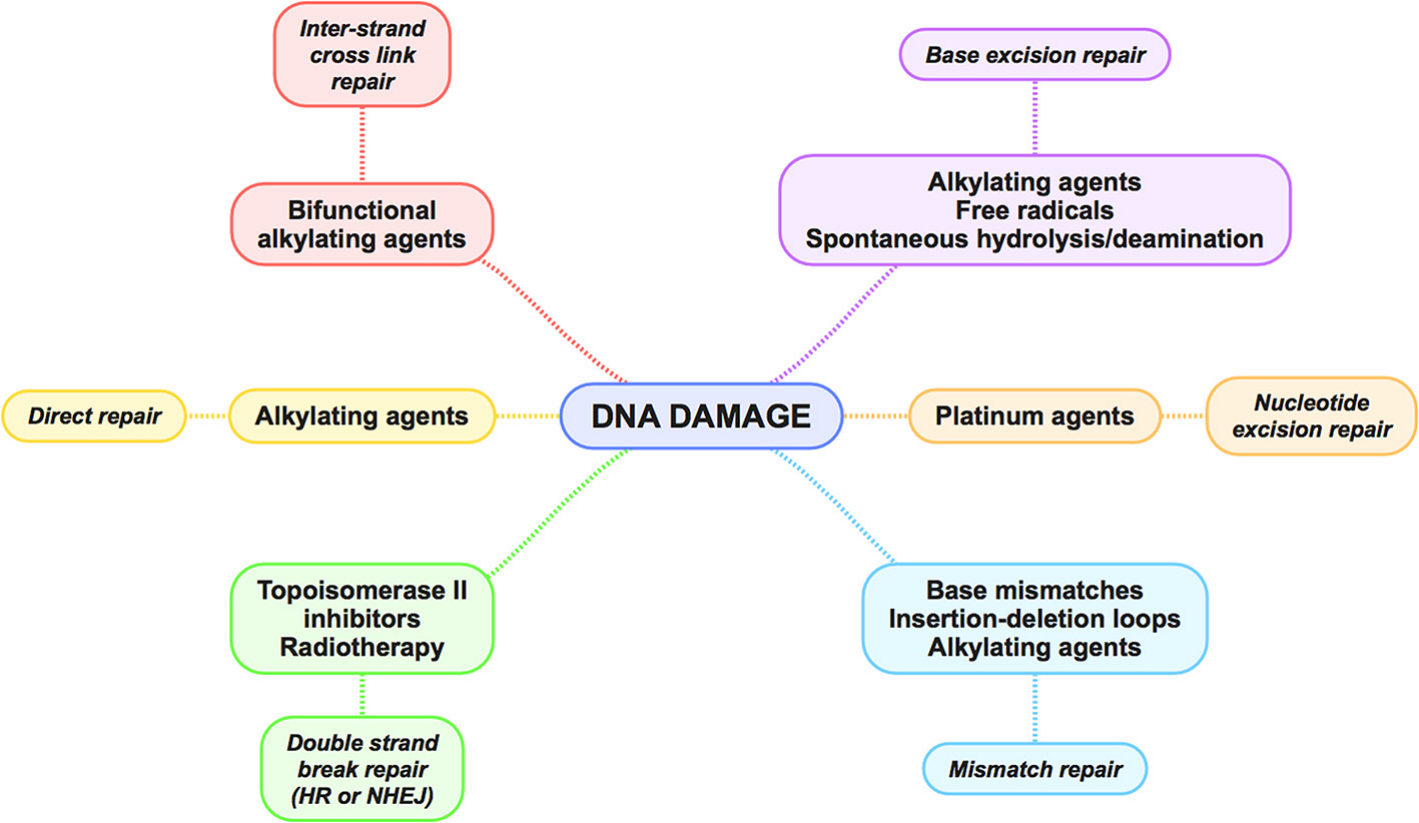
An overview of mammalian DNA repair pathways

DNA repair in mammalian cells occurs through six key pathways, dependent on the type of DNA lesion, which are briefly outlined here and are more comprehensively reviewed elsewhere [3]. Figure 2 highlights the potential repair pathways for different types of DNA damage. It should be noted that significant cross-over exists between the effector proteins in each pathway.

#### Direct reversal

A small subset of DNA lesions, namely UV and alkylation-induced damage, can be directly reversed *in situ* in a relatively ‘error-free’ manner, without cleavage of the DNA phosphodiester backbone. UV radiation results in DNA photolesions including cyclobutane pyrimidine dimers (CPDs) and pyrimidine (6–4) pyrimidine photoproducts that are repaired by specific photolyases [4]; this direct repair occurs primarily in prokaryotes, whereas human cells repair these lesions by nucleotide excision repair (NER) [5]. Various alkylating lesions can occur following treatment with alkylating agents, a commonly used class of chemotherapy drug [6]. While some alkylating lesions may be repaired by base excision repair (BER), direct reversal can be performed by the sacrificial enzymes O^6^-alkylguanine-DNA alkyltransferase (AGT) or methylguanine methyltransferase (MGMT) [7]. Levels of MGMT in tumors, therefore, partially determines the response to alkylating chemotherapy agents [8,9]. An alternative pathway is the oxidative reversal of alkylation damage by AlkB dioxygenases (ABH2 and ABH3) [7,10].

#### BER

The BER pathway has evolved to detect and repair DNA damage from ROS, spontaneous deamination, and exogenous causes including alkylating agents and ionizing radiation [11]. BER can be further classified into short-patch (single nucleotide) and long-patch (multiple nucleotide) repair but the overall pathway is similar for both [12]. The BER pathway has five key steps: (i) removal of the base at the site of damage by a specific DNA glycosylase creating an apurinic/apyrimidinic (AP) site; (ii) incision of the phosphodiester backbone at the AP site by AP-endonuclease 1 (APE1); (iii) removal of the remaining deoxyribose-phosphate fragment by either DNA polymerase-β (polβ) (short-patch) or flap-endonuclease 1 (FEN1) (long-patch); (iv) insertion of the correct base at the AP site by DNA polymerases, predominantly by polβ (facilitated by X-ray cross-complementing group 1 protein (XRCC1)), but also polδ and ε (mediated by proliferating cell nuclear antigen (PCNA)) and (v) resealing the incised strand by DNA ligases [12,13]. Single-strand DNA breaks (SSBs), such as those made by APE1, cause the activation of poly(ADP-ribose) polymerase 1 (PARP1), which accelerates the repair pathway by recruiting essential BER enzymes such as DNA polymerases, ligases and XRCC1 [14,15]. PARP2 also plays a role in BER; however, neither PARP enzyme is essential for successful BER as it can proceed in their absence [16].

In contrast, PARP enzymes play a vital role in single-strand break repair (SSBR), an important subpathway of BER. SSBs can result from ROS, base deamination, intermediates from BER, defective activity of DNA topoisomerase 1 or replication-associated damage [3,17]. In summary, SSBR has four key steps: (i) SSB detection by PARP1 that adds PAR to itself (autoPARylation) and other repair proteins while also recruiting XRCC1 before dissociating from DNA; (ii) processing of the damaged 3′ or 5′ termini of the damaged strand by APE1, polβ, and polynucleotide kinase/phosphatase (PNKP) in conjunction with XRCC1, or in some cases by FEN1 in conjunction with PCNA; (iii) DNA gap filling with nucleotide insertion, and (iv) DNA ligation.

#### NER

The NER pathway is responsible for recognizing and repairing DNA lesions that cause a significant distortion of its helical structure [18]. Two subpathways of NER exist: transcription-coupled NER (TC-NER) for actively transcribed DNA and global-genome NER (GG-NER) for nonactively transcribed DNA. In summary, both NER pathways have the following steps: (i) recognition of DNA damage through sensor proteins [19,20]; (ii) recruitment of transcription factor IIH that contains helicases XPB and XPD to unwind DNA surrounding the lesion; (iii) incisions on either side of the lesion are made by the endonucleases XPG and ERCC1-XPF complex to produce an oligonucleotide product of 25–30 nucleotides in length; and (iv) polε in combination with PCNA and ligase I (in replicating cells) and pol δ and κ with PCNA and ligase IIIα/XRCC1 (in quiescent cells) act to fill and seal the incised gap [2,20]. Germline mutations in components of the NER pathway are a known cause of xeroderma pigmentosum in which patients have a greater than 1000-fold increased risk of nonmelanoma skin cancer [21].

#### Mismatch repair

The mismatch repair (MMR) pathway is responsible for the recognition and repair of base–base mismatches and insertion/deletion loops (IDLs) that form during DNA replication. Errors that evade the intrinsic proofreading activity of DNA polymerases must be corrected by the MMR pathway [22]. Additionally, MMR is responsible for correction of IDLs within microsatellite DNA and consequently, defective MMR results in ‘microsatellite instability,’ which can in-turn lead to genomic instability. In eukaryotes, MMR is commenced by the MSH2-MSH6 (small mismatches) or MSH2-MSH3 (large mismatches or IDLs) complexes. Both complexes then share a common pathway by first recruiting the MLH1-PMS2 complex which, in conjunction with PCNA, clamps to the DNA lesion [23]. These complexes now work in conjunction with exonuclease-1 (EXO1), polδ and DNA ligase I to excise and reform DNA using the other strand as a template [3]. There is evidence that FEN1 may act as an exonuclease in the MMR pathway, highlighting the cross-over of proteins between different repair pathways [24]. Mutations in the MMR pathway, most notably in MSH2 and MLH1, predispose to cancer and are an identified cause of hereditary nonpolyposis colorectal cancer (HNPCC) [25].

#### Nonhomologous end-joining

Double-strand breaks (DSBs) may occur as a result of ionizing radiation, ROS, replication errors or in physiological circumstances, such as V(D)J recombination for developing adaptive cell immunity, and certain chemotherapeutics. DSBs are either repaired by the more error-prone (and hence mutagenic) nonhomologous end-joining (NHEJ) or by the more accurate homologous recombination (HR). In brief, NHEJ occurs as follows: (i) recognition of a DSB by the Ku complex; (ii) Ku recruits DNA-dependent protein kinase catalytic subunit (DNA-PKcs) to form a DNA-PK complex; (iii) broken end processing (if necessary) is conducted by endonucleases; (iv) nucleotide insertion is performed by DNA polymerases λ and μ, which bind to Ku via their N-terminal BRCA1 C terminus (BRCT) domains; and (v) ligation occurs via the DNA ligase IV and XRCC4 complex [26,27]. Polymerase activity in NHEJ can be either template-dependent or independent, the latter being a key source of error in the pathway, and more commonly performed by polμ. Although often labelled as ‘error-prone’, DSBs resulting in blunt ends can be repaired with high precision by NHEJ, although such damage does not typically occur with ionizing radiation. Defects in the NHEJ pathway therefore lead to greater sensitivity to ionizing radiation, due to an inability to repair DSBs [28].

Microhomology-mediated end joining (MMEJ) and single-strand annealing (SSA) are mutagenic NHEJ-related pathways that have been reviewed in more detail elsewhere [3,29,30] and are beyond the scope of this review. It is relevant to note that MMEJ may be initiated by PARP1 [29] and is facilitated by polθ [31].

#### HR

The HR pathway utilizes a homologous template DNA strand to ensure highly accurate repair of DSBs and DNA interstrand cross-links (ICLs). The choice of which DSB repair pathway to follow is dependent on stage of the cell cycle (HR is up-regulated during S and G2 due to availability of a template strand) [32] and type of DSB sustained, with more complex breaks and those occurring during replication preferentially repaired by HR [33]. In summary, HR has the following steps: (i) damage recognition and resection of damaged ends by the MRN complex (Mre11-Rad50-Nbs1) producing single-strand DNA (ssDNA); (ii) coating of ssDNA by Replication Protein A (RPA); (iii) BRCA2-mediated replacement of RPA with RAD51; (iv) RAD51-bound ssDNA searches for and invades the homologous sequence on the sister chromatid; (v) repair synthesis occurs using the template strand by polη; (vi) dissociation of the repaired strand from the template strand followed by (vii) end ligation. These final resolution stages can either occur through synthesis-dependent strand annealing (SDSA) or through formation of Holliday junctions, which may generate cross-over products [33]. Both pathways are reviewed in more detail elsewhere [3,34]. Notably, germline mutations in BRCA2 result in greater susceptibility to breast, ovarian, and other cancers, evidencing the importance of HR in the maintenance of genomic integrity [35].

A subset of HR involves the repair of ICLs; these are recognized and corrected by a range of effector proteins including the Fanconi Anaemia (FA) complex, BRCA1, polν, and other proteins involved in HR including RAD51. While cross-link repair is reviewed in [36], it should be noted that up-regulation of ICL repair proteins may be responsible for resistance to platinum agents, whose primary mechanism of action is DNA damage through creation of ICLs. Consequently, the ICL repair pathway may offer a novel therapeutic target, with the aim of restoring platinum sensitivity.

### DNA repair and cancer

If DNA lesions caused by the aforementioned agents remain unrepaired, mutations may arise which, in turn, can promote neoplastic transformation and subsequent carcinogenesis. As noted above, germline mutations in DNA repair proteins are recognized causes of hereditary cancer syndromes. The ‘mutator phenotype’ suggests that an impairment of one or more DNA repair pathways significantly promotes mutagenesis. Based on selection pressures, mutations in tumor-suppressor genes and oncogenes are more favorable to cell survival and growth [37]. Even following carcinogenesis, alterations in certain DNA repair pathway proteins have been shown to correlate with more aggressive tumors and consequently, worse prognosis [38,39].

**Figure 3:**
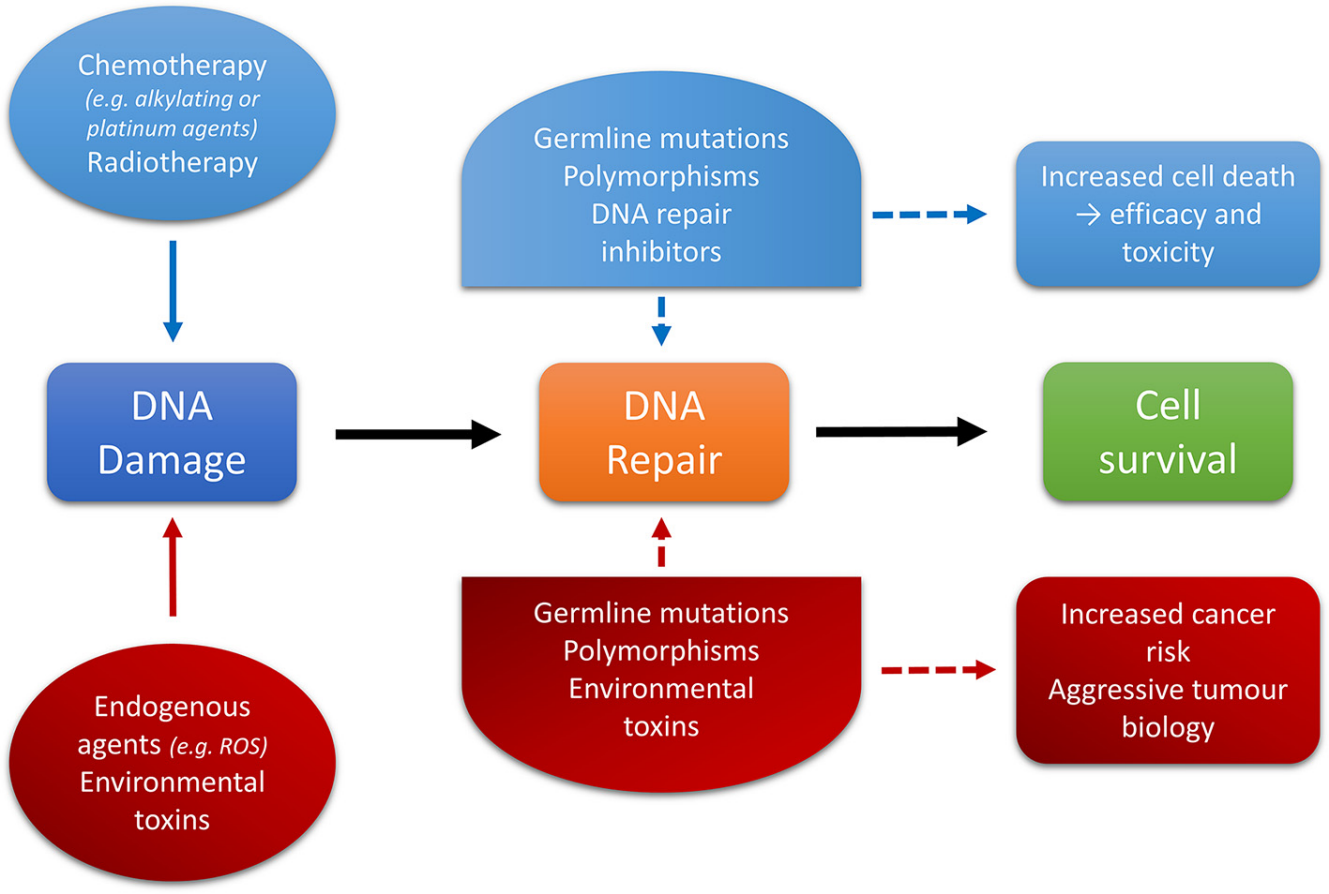
DNA repair and cancer, a complex interaction

The underlying mechanism of action of many chemotherapeutic agents and therapeutic ionizing radiation is primarily through initiation of DNA damage, with the aim of inducing cell cycle arrest or apoptosis of cells within the tumor. It therefore follows that intact or even up-regulated DNA repair pathways may contribute to treatment resistance [39]. This complex relationship between DNA repair, carcinogenesis, and therapeutic response is outlined in Figure 3.

Based on this rationale, inhibition of DNA repair pathways should potentiate the cytotoxic effects of chemotherapy and radiotherapy by acting as ‘sensitizing agents’ and overcoming resistance. Theoretically, such combinations should improve fractional cell kill and, by extension, clinical effectiveness. However, impairing DNA repair in other rapidly proliferating tissues, such as bone marrow and gastrointestinal mucosa, will result in increased toxicity to these cells and potential life-threatening complications, limiting the utility of such combinatorial therapeutic approaches [40,41]. This is evidenced clinically in trials of MGMT inhibitors and more recently, PARP inhibitors (PARPi) and other DNA repair inhibitors, in combinations with chemotherapy [41–43].

One means of overcoming this challenge is through more localized targeting of the cytotoxic component of combination therapies, which can be achieved with radiotherapy. While preclinical and clinical effectiveness of DNA repair inhibitors and radiotherapy in combination has been demonstrated, concerns remain regarding potential toxicity to normal tissues [44,45].

As a result, there is now a greater emphasis on developing personalized, precision treatments, which can selectively damage tumor cells and minimize toxicity to normal tissues. One means of achieving this is through targeting tumor-specific alterations in DNA repair pathways, thereby exploiting the concept of ‘synthetic lethality.’ In the present review, we will discuss current and evolving synthetic lethality targets in cancer therapy.

### Synthetic lethality

**Figure 4:**
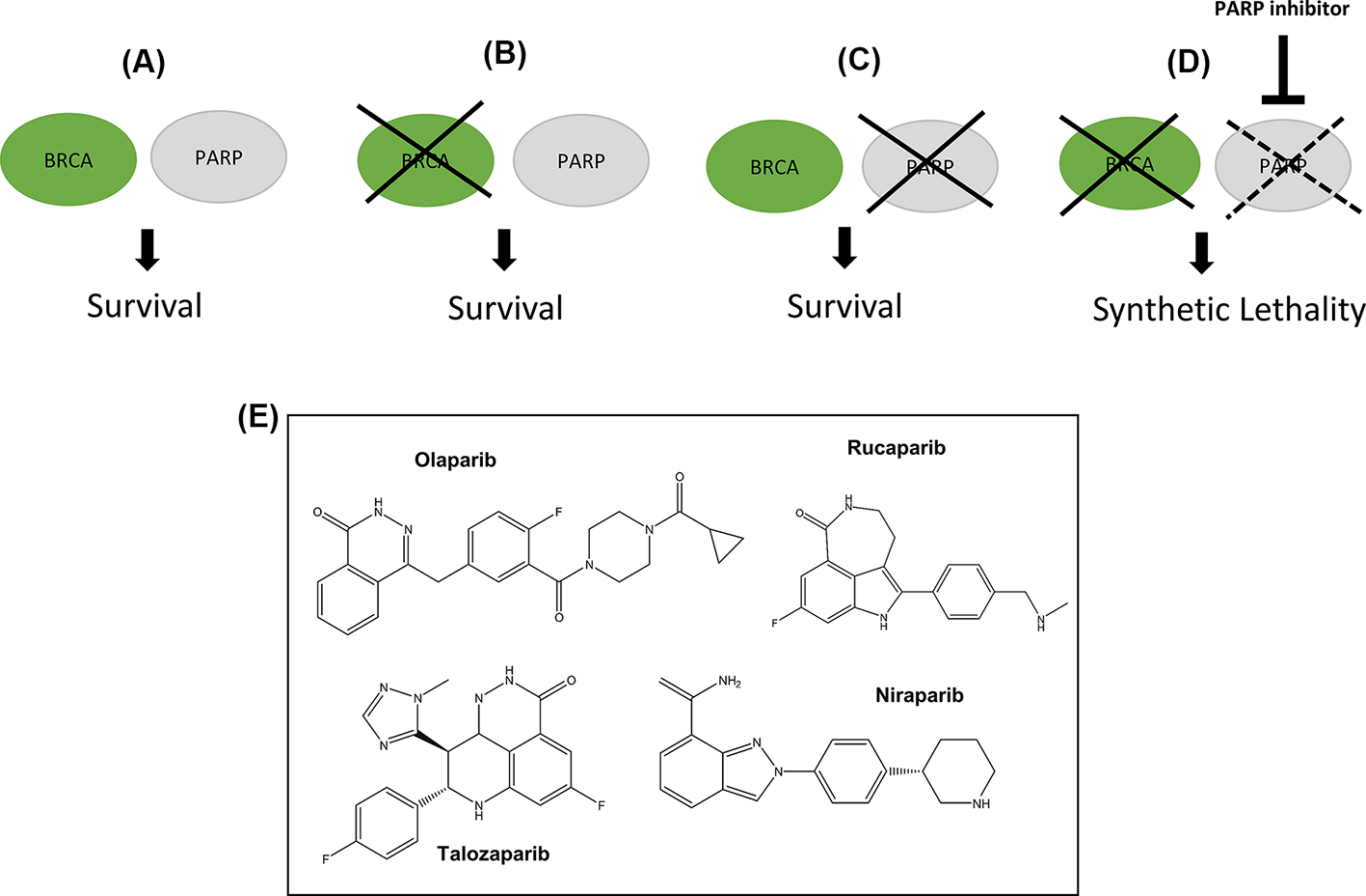
Synthetic lethality

Synthetic lethality refers to the situation in which a loss of function of either one of two genes does not result in cell death (and may even confer a survival advantage), whereas loss of both genes results in cell death [46] (Figure 4A–D). DNA repair inhibitors can theoretically capitalize on this principle. Tumors often harbor mutations in one or more DNA repair pathways, leading to a reliance on alternative, functioning pathways. Therefore, inhibition of an important alternative pathway can lead to a nonviable accumulation of unrepaired DNA damage (from constant endogenous damage, chemotherapy, or radiotherapy), and subsequent apoptosis. Normal cells possess an intact pathway to repair such damage, leading to selective killing of cancer cells.

### PARP and BRCA

The discovery that PARP1 inhibition can lead to selective killing of BRCA-mutant cells has formed the cornerstone of evidence for synthetic lethality strategies. As outlined above, PARP plays an essential role in SSBR. Some PARPi, such as olaparib, rucaparib, niraparib, and talazoparib (chemical structures shown in Figure 4E), act by ‘trapping’ PARP1 at its binding site on DNA, thus inhibiting autoPARylation and PARP1 dissociation. The trapped PARP1 protein is cytotoxic (rather than the unrepaired SSB), as it causes collapse of the replication fork leading to a DSB [47]. In ‘normal’ cells, these breaks are repaired by the HR pathway, while in BRCA-deficient cells they remain unrepaired and accumulate, eventually resulting in apoptosis. However, these PARPi vary in their ‘trapping’ efficacy while veliparib, a different PARPi, acts primarily through inhibition of autoPARylation rather than ‘trapping’ [46]. This therefore highlights the heterogeneity of currently available PARPi and may partially explain their differing toxicity profiles [48].

Following promising preclinical evidence supporting this synthetically lethal interaction [49,50], PARPi have been investigated in a number of clinical trials for treating BRCA-mutated cancers. It is estimated that 5–10% of breast and ovarian cancers carry either germline or somatic BRCA mutations [51]. A summary of phase III trials of PARPi for BRCA-mutated cancers is presented in Table 1 [52–61]. These trials in BRCA-mutated breast cancers have demonstrated that olaparib [59], talazoparib [58], and veliparib [54], either as monotherapy or in combination with chemotherapy, significantly improved median progression-free survival (mPFS) as compared with standard chemotherapy regimens alone. These findings are further supported by preceding phase II trials that have also demonstrated acceptable toxicity profiles [62,63]. Consequently, olaparib and talazoparib are licensed in the U.S.A. for the treatment of BRCA-mutated, HER2-negative breast cancers, although approval is still awaited in the U.K. PARPi have been most extensively investigated in the treatment of BRCA-mutated ovarian cancers as both combination therapies and as monotherapy. All published phase III trials have demonstrated significant benefits in mPFS in favor of PARPi, with acceptable toxicity [52,53,55,60]. Based on this evidence, olaparib and niraparib have been approved by the FDA, and NICE in the U.K., for the treatment of BRCA-mutated, advanced ovarian cancer. In addition, a phase III trial of olaparib for the treatment of BRCA-mutated advanced pancreatic cancer demonstrated significantly greater mPFS for PARPi as compared with placebo (7.4 vs 3.8 months; *P*=0.004), resulting in its FDA approval for this indication [57].

**Table 1:** Published phase III trials of PARPi for BRCA-mutated cancers

Study title	Year published	Cancer	Author	PARPi	Comparator arm	Sample size	mPFS	Other relevant results
Rucaparib versus standard-of-care chemotherapy in patients with relapsed ovarian cancer and a deleterious BRCA1 or BRCA2 mutation (ARIEL4): an international, open-label, randomised, phase 3 trial [50]	2022	Ovarian	Kristeleit et al.	Rucaparib (600 mg BD)	Chemotherapy	349	Rucaparib 7.4 months vs chemotherapy 5.7 months (*P*=0.001)	NA
Maintenance olaparib for patients with newly diagnosed advanced ovarian cancer and a BRCA mutation (SOLO1/GOG 3004): 5-year follow-up of a randomised, double-blind, placebo-controlled, phase 3 trial [51]	2021	Ovarian	Banerjee et al.	Olaparib (300 mg BD)	Placebo	391	Olaparib 56.0 months vs placebo 13.8 months	NA
Veliparib with carboplatin and paclitaxel in BRCA-mutated advanced breast cancer (BROCADE3): a randomised, double-blind, placebo-controlled, phase 3 trial [52]	2020	Breast	Diéras et al.	Veliparib (120 mg BD) plus chemotherapy	Placebo plus chemotherapy	513	Veliparib plus chemotherapy 14.5 months vs placebo plus chemotherapy 12.6 months (*P*=0.0016)	NA
Olaparib versus nonplatinum chemotherapy in patients with platinum-sensitive relapsed ovarian cancer and a germline BRCA1/2 mutation (SOLO3): a randomized phase III trial [53]	2020	Ovarian	Penson et al.	Olaparib (300 mg BD)	Physician’s choice single-agent nonplatinum chemotherapy	266	Olaparib 13.4 months vs physician choice chemotherapy 9.2 months (*P*=0.013)	ORR significantly higher for olaparib (72.2% vs 51.4%)
Veliparib with first-line chemotherapy and as maintenance therapy in ovarian cancer [54]	2019	Ovarian	Coleman et al.	Veliparib (150 mg OD) plus chemotherapy followed by veliparib maintenance	Chemotherapy plus placebo, chemotherapy plus veliparib followed by placebo maintenance	1140	Veliparib maintenance 23.5 months vs chemotherapy plus placebo 17.3 months (*P*<0.001)	In BRCA-positive group, mPFS: 34.7 months for veliparib maintenance vs 22.0 months for placebo. In HR defect group, mPFS: 31.9 months for veliparib maintenance vs 20.5 months for placebo (*P*<0.001 for both)
Maintenance olaparib for germline BRCA-mutated metastatic pancreatic cancer [55]	2019	Pancreatic	Golan et al.	Olaparib (300 mg BD)	Placebo	154	Olaparib 7.4 months vs placebo 3.8 months (*P*=0.004)	Median overall survival: olaparib 18.9 months vs placebo 18.1 months (*P*=0.68)
Talazoparib in patients with advanced breast cancer and a germline BRCA mutation [56]	2018	Breast	Litton et al.	Talozaparib (1 mg OD)	Chemotherapy	431	Talozaparib 8.6 months vs chemotherapy 5.6 months (*P*<0.001)	ORR: talozaparib 62.6% vs chemotherapy 27.2%
Olaparib for metastatic breast cancer in patients with a germline BRCA mutation [57]	2017	Breast	Robson et al.	Olaparib (300 mg BD)	Chemotherapy	302	Olaparib 7.0 months vs chemotherapy 4.2 months (*P*<0.001)	ORR: olaparib 59.9% vs chemotherapy 28.8%
Olaparib tablets as maintenance therapy in patients with platinum-sensitive, relapsed ovarian cancer and a BRCA1/2 mutation (SOLO2/ENGOT-Ov21): a double-blind, randomised, placebo-controlled, phase 3 trial [58]	2017	Ovarian	Pujade-Lauraine et al.	Olaparib (300 mg BD)	Placebo	295	Olaparib plus bevacizumab 19.1 months vs placebo 5.5 months (*P*<0.0001)	NA
Niraparib maintenance therapy in platinum-sensitive, recurrent ovarian cancer [59]	2016	Ovarian	Mirza et al.	Niraparib (300 mg OD)	Placebo	553	In gBRCA cohort: 21.0 months for niraparib vs 5.5 months placebo. In non-BRCA group: 9.3 months for niraparib vs 3.9 months placebo (*P*<0.001 for both)	For non-BRCA group with HR deficiency, mPFS: 12.9 months with niraparib vs 3.8 months with placebo (*P*<0.001)

Further to these promising findings, PARPi are also effective preclinically in tumors that do not possess a BRCA mutation but are deficient in another component of the HR pathway, thereby exploiting an alternative synthetically lethal pairing [64]. Such HR-deficient tumors are described as demonstrating ‘BRCAness’ with commonly affected genes including ATM, ATR, PALB2, CHEK1 (encoding CHK1), and CHEK2 [65]. Somatic mutations in these genes, in keeping with the mutator phenotype, are widespread across many cancer types. These cancers, as well as those harboring a BRCA mutation, typically possess a characteristic series of mutations due to an over-reliance on more error-prone repair pathways. Such mutational scars can be identified using mutational signature profiles to assist in distinguishing those most suitable for PARPi therapy. In clinical trials, PARPi have been shown to significantly improve mPFS in HR-deficient ovarian cancers [56,61] with promising results seen in HR-deficient advanced prostate [66,67] and urothelial [68] cancers. Beyond this, Coleman et al. demonstrated that addition of veliparib to chemotherapy in advanced ovarian cancer significantly improved mPFS as compared with chemotherapy and placebo (23.5 vs 17.3 months; *P*<0.001) even in patients without BRCA or known HR repair mutations [56].

However, developing resistance to PARPi is thought to occur in 40–70% of patients over the course of their treatment [69]. The most notable mechanism, and the only one as yet confirmed *in vivo*, is a frameshift mutation in BRCA2 that restores the open-reading frame of the gene, thereby restoring its function without a complete reversal back to wild-type [70,71]. This *c.6174delT* frameshift mutation will result in restored HR repair capabilities and prevention of synthetic lethality in the presence of PARPi [69]. Other mechanisms of resistance occur through an inhibition of NHEJ, thereby forcing cells to repair DSBs via the HR pathway. First, p53-binding protein 1 (53BP1) is a key repair protein that, in BRCA-mutant cells, typically inhibits HR and drives excessive NHEJ and consequent apoptosis. In normal situations, 53BP1 is removed by BRCA1 to allow for repair by the more accurate HR pathway. As a result, loss of 53BP1, as often occurs in BRCA-mutated or triple-negative breast cancers, results in partial restoration of the HR pathway and resistance to PARPi. Evidence suggests that 53BP1 levels may act as a prognostic biomarker by predicting response to both chemotherapy agents and PARPi [69]. Second, the REV7 protein acts in a similar manner, but downstream of 53BP1, by promoting DSB repair via the NHEJ pathway. Consequently, reduced levels of REV7 promote HR repair, hence conferring PARPi resistance and worse patient outcomes [69,72]. More recent evidence suggests that PARPi resistance may occur through restoring fork stabilization via loss of proteins such as PTIP, RADX, SMARCAL1, and FANCD2; this is reviewed more extensively in [69]. Another preclinical mechanism for PARPi resistance is increased expression of ABCB1 that encodes a drug transporter, resulting in increased efflux of PARPi [73]. Finally, mutations or reduced levels of PARP1 itself may lead to resistance to PARPi due to a loss of the cytotoxic ‘trapped’ PARP1 at sites of SSBs [69].

Overcoming PARPi resistance has led to a need to identify new synthetically lethal pairings and novel targets for DNA repair inhibition. Recent evidence suggests that potential targets include proteins involved in cell-cycle checkpoints and DNA repair pathways such as ATR, ATM, and WEE1.

### ATR/CHK1

The ataxia telangiectasia and Rad3-related (ATR)-checkpoint kinase 1 (CHK1) pathway is a key component of the DNA damage response. Following end resection of DSBs (as mediated by ATM) or at stalled replication forks, ssDNA is coated by RPA as discussed above. This bound RPA subsequently recruits ATR and ATR interacting protein (ATRIP) that in turn activates CHK1. CHK1 then acts to inhibit CDK2 during the S-phase by causing degradation of CDC25A. The reduced activity of CDK2 results in activation of the intra-S and G2/M phase cell-cycle checkpoints, allowing the cell to initiate DSB repair. Furthermore, downstream components of the ATR-CHK1 pathway play a key role in suppressing the replication stress response, which is triggered by stalled replication forks [74].

The ATR/CHK1 pathway has been identified as a potential synthetic lethality target. Cells that lack a functional G1 checkpoint, as often occur in tumors with p53 or retinoblastoma mutations, may be particularly sensitive to ATR/CHK1 inhibition. In such cells, ATR/CHK1 inhibition will result in loss of the G1, intra-S and G2/M checkpoints with premature progression to mitosis leading to a ‘mitotic catastrophe’ and cell death [74]. As discussed above, 53BP1 mutations may lead to PARPi resistance and ATR inhibition may therefore offer a means of overcoming this. Similarly, chemotherapy agents trigger a replication stress response as a result of DNA damage; inhibition of ATR will prevent suppression of this response and in tumor cells that overexpress oncogenes, this can be synthetically lethal [74].

ATR inhibitors (ATRi) currently in use are predominantly small-molecule inhibitors that preclinically have been shown to sensitize cells to ionizing radiation and chemotherapy, as well as inducing synthetic lethality in p53- and ataxia telangiectasia mutated (ATM)-deficient cell lines. Furthermore, ATR/CHK1 inhibition sensitized cells to PARPi, thereby rationalizing combination strategies [75].

Following these promising *in vitro* results, four ATRi have been used in clinical trials, namely M6620 (berzosertib, IV), AZD6738 (PO), BAY1895344 (PO), and most recently RP-3500 (PO). It should be noted that ATRi, particularly in combination with other chemotherapy agents, carry a significant toxicity profile with over a third of patients across two trials experiencing grade 3 or 4 adverse events, predominantly cytopenias [76,77]. M6620 has shown some promise as a monotherapy with pre- and post-treatment tumor biopsies demonstrating a reduction in CHK1 phosphorylation (a biomarker of ATR activity) [78]. In phase I trials, ATRi have been shown to act synergistically with chemotherapy agents such as platinum agents [76–78], gemcitabine [79], and topotecan [80]. The phase I Patriot study investigating the combination of ATR inhibition with ionizing radiation is ongoing, although there is positive preclinical evidence [74]. To date, there are no published phase II clinical trials evaluating ATRi as they remain in the early stages of development; ongoing phase II trials of ATRi are summarized in Table 2.

**Table 2:** Ongoing phase II trials of ATRi

Study title	NCT trial number	Year commenced	Cancer(s)	ATRi	Comparator	Estimated/ actual enrollment	Specific biomarkers	Estimated study completion date
ATARI: ATr inhibitor in combination with olaparib in gynaecological cancers with ARId1A loss	NCT04065269	2019	Gynecological cancers (including ovarian and endometrial)	AZD6738 plus olaparib	AZD6738 alone	105	ARID1A	March 2023
Phase II trial of AZD6738 alone and in combination with olaparib in patients with selected solid tumor malignancies	NCT03682289	2019	Advanced solid tumors (excluding ovarian cancer)	AZD6738 plus olaparib	AZD6738 alone	59	BAF250a and ATM	December 2023
Phase 1/2a study of the safety, pharmacokinetics, pharmacodynamics and preliminary clinical activity of RP-3500 alone or in combination with talazoparib or gemcitabine in advanced solid tumors with ATR inhibitor sensitizing mutations (TRESR study)	NCT04497116	2020	Advanced solid tumors	RP-3500	RP-3500 plus talazoparib plus gemcitabine	451	Not specified	March 2024
A multi-center phase II study testing the activity of olaparib and AZD6738 (ATR inhibitor) in metastatic castration-resistant prostate cancer	NCT03787680	2019	Prostate	AZD6738 plus olaparib	Nil	49	NA	November 2026
Phase 1b/2 study of ATR inhibiTor RP-3500 and PARP inhibitor combinations in patients with molecularly selected cancers (ATTACC)	NCT04972110	2021	Advanced solid tumors	RP-3500 plus niraparib or olaparib	Nil	108	Not specified	November 2023
Phase 2 study of M6620 (VX-970) in combination with gemcitabine versus gemcitabine alone in subjects with platinum-resistant recurrent ovarian or primary peritoneal fallopian tube cancer	NCT02595892	2016	Ovarian	M6620 (berzosertib) plus gemicitabine	Gemcitabine alone	70	Nil	March 2023
A phase I/II trial of lurbinectedin with berzosertib, an ATR kinase inhibitor in small cell cancers and high grade neuroendocrine cancers	NCT04802174	2021	Small-cell lung cancer and high-grade neuroendocrine tumors	M6620 (berzosertib) plus lurbinectedin	Nil	75	Nil	December 2026
A phase I/II trial of topotecan with VX-970 (M6620), an ATR kinase inhibitor in small-cell cancers	NCT02487095	2015	Small-cell cancers (lung and extrapulmonary)	M6620 (berzosertib) plus topotecan	Nil	62	Nil	October 2025
A phase I/IIa, open-label, multi-center study to assess the safety, tolerability, pharmacokinetics and preliminary efficacy of the ATR kinase inhibitor ART0380 administered orally as monotherapy and in combination to patients with advanced or metastatic solid tumors	NCT04657068	2021	Advanced solid tumors with ATM mutation or ovarian cancer	ATR0380 plus gemcitabine or irinotecan	ART0380 alone	232	ATM	December 2023
A phase 1/ 2 study of BAY 1895344 (Elimusertib, NSC#810486) in pediatric patients with relapsed or refractory solid tumors	NCT05071209	2021	Relapsed solid tumors	Elimusertib	Nil	23	ATM, ATRX, BRCA1, BRCA2, CDK12, CHEK1, CHEK2, FANCA, MSH2, MRE11, PALB2, PARP1, POLD1, RAD51, or XRCC2	June 2024
National lung matrix trial: multi-drug, genetic marker-directed, non-comparative, multi-centre, multi-arm phase II trial in non-small cell lung cancer	NCT02664935	2015	Non-small-cell lung cancer	AZD6738 plus durvalumab	AZD4547, vistusertib, palbociclib, crizotinib, selumitinib plus docetaxel, AZD5363, osimertinib, durvalumab, sitravatinib	423	KRAS, SKT11/LKB1	October 2022
Combination ATR and PARP inhibitor (CAPRI) trial with AZD 6738 and olaparib in recurrent ovarian cancer	NCT03462342	2018	Recurrent ovarian cancer	AZD6738 plus olaparib	Nil	86	BRCA or HRD mutations	December 2022

Preclinical evidence suggests that ATR inhibition may lead to down-regulation of programmed death-ligand 1 (PDL-1), thereby sensitizing tumors to immune-cell mediated killing. Consequently, a phase I study evaluated the safety and effectiveness of AZD6738 combined with the PDL-1 inhibitor durvulumab; the combination was well tolerated and promisingly, there was one potential complete response and one partial response in the cohort [76].

On the basis of *in vitro* evidence suggesting that ATRi can sensitize cancer cells to PARPi treatment [81], a number of trials testing this combination have commenced. Yap et al. assessed the combination of AZD6738 and olaparib in a phase I trial in which two patients with triple-negative breast cancer (TNBC) achieved partial responses.

Mechanisms of resistance to ATRi continue to be investigated; Lloyd et al. identified using CRISPR–Cas9 genome-wide screening that loss of cyclin C or CDK8 can lead to ATRi resistance through suppression of the replication stress response [82]. Further elucidation of these resistance mechanisms, particularly in *in vivo* models, is essential to identify patients mostly likely to respond and therefore develop more successful targeted, precision therapies.

Selective CKH1 inhibitors, such as MK-8776, have shown positive results in preclinical studies as monotherapy [83], with chemotherapy agents [84] and with ionizing radiation [85]. MK-8776 has been well tolerated in trials with gemcitabine and cytarabine for solid tumors (phase I) [86] and refractory acute leukemias (phase II) [87], respectively. There was promising antitumor activity in solid tumors [86], while for hematological malignancies, the addition of MK-8776 to cytarabine had no significant benefit [87]. Prexasertib, a second-generation CHK1 inhibitor with some anti-CHK2 activity, has been investigated in phase II trials for ovarian cancer [88] and TNBC [89] with encouraging results, although severe neutropenia was common in both. Further trials investigating CHK1 inhibitors across various tumor types are ongoing. Intriguingly, recent evidence suggests CHK1 inhibitors may induce BRCAness in cells, thereby sensitizing BRCA wild-type but p53-deficient cells to olaparib. This rationalizes combinations of CHK1 inhibitors and PARPi and hence warrants further investigation in clinical trials [90].

### ATM/CHK2

ATM/CHK2 signaling also plays an essential role in the DNA damage signaling response and repair (DDR), in particular the recognition and repair of DSBs. As discussed above, DSBs are recognized by the MRN complex; this complex subsequently activates ATM-CHK2 kinase resulting in phosphorylation of p53 and cell-cycle arrest at the G1/S checkpoint. Furthermore, ATM mediates end processing of DSBs resulting in RPA coating of ssDNA and therefore activation of the ATR/CHK1 pathways as described above. ATM and both ATR and p53 demonstrate a synthetically lethal relationship as the loss of three key cell-cycle checkpoints results in mitotic catastrophe. Although ATM is frequently mutated across cancer types, functional ATM deficiency due to hypermethylation of its promoter region is more common [91]. Preclinical evidence suggests that ATM activation may contribute to chemotherapeutic resistance [92]. This therefore rationalizes the development of ATM inhibitors (ATMi; and downstream CHK2 inhibitors) both as a means of overcoming chemotherapy resistance, sensitizing cells to ionizing radiation and as a synthetic lethality strategy in p53-deficient tumors.

In the laboratory setting, a wide range of ATMi have been tested. These studies have demonstrated that ATMi can sensitize cells to chemotherapy and ionizing radiation, although they appear to lack utility as a monotherapy. Phosphate and tensin homolog (PTEN) plays an important role in the DDR. Multiple preclinical studies across tumor types have demonstrated that the ATMi KU-60019 in combination with cisplatin is synthetically lethal in PTEN-deficient cells [93,94].

While these studies demonstrate the potential benefits of ATM inhibition, the development of ATMi for clinical studies remains in its infancy. Three ATMi (AZD0156, KU-60019, and AZD1390) are being investigated in clinical trials with results expected in the coming years. AZD0156 is being investigated as monotherapy, in conjunction with other chemotherapy agents, and as a combination therapy with olaparib for the treatment of advanced solid tumors (NCT02588105). AZD1390 is being assessed in combination with ionizing radiation for the treatment of glioblastoma multiforme (NCT034236280). Finally, KU-60019 is being assessed in combination with silimitasertib (a casein kinase II inhibitor involved in the PI3K/AKT pathway) for the treatment of renal cell cancers (NCT03571438) [95]. Further preclinical research to identify potential mechanisms of resistance to ATMi will be a vital aspect of their ongoing development.

As a downstream target of ATM, CHK2 inhibitors can also induce mitotic catastrophe in a similar manner to CHK1 inhibition. In preclinical studies, two selective CHK2 inhibitors have been investigated: PV1019 (NIH) and CCT241533 (ICR). The former has been shown to act synergistically with chemotherapy and radiotherapy [96], while the latter can potentiate the activity of PARPi [97]. While these selective inhibitors have not, as yet, been investigated in clinical trials, the nonselective CHK1/2 inhibitor AZD7762 has undergone phase I trials with promising results. However, AZD7762 was associated with significant, dose-limiting cardiotoxicity [98] and two other phase I trials with the drug were suspended (NCT00937664 and NCT00473616). Additional research into the toxicity profile of CHK inhibitors is necessary in order to determine whether the observed cardiotoxicity is a wider problem with the whole drug class.

### WEE1

WEE1 kinase plays an essential role at the G2/M cell-cycle checkpoint. The enzyme acts by phosphorylating, and thereby inhibiting, CDK1/cyclin complexes and hence preventing cell-cycle progression to mitosis [99]. In addition to controlling cell-cycle progression and maintaining genomic integrity, WEE1 also plays a role in epigenetic modulation through suppression of histone transcription in late S-phase [100]. WEE1 expression has been shown to be both up- and down-regulated across different cancer types, with both associated with poor prognosis [101]. In those tumors with high WEE1 expression, it is likely that they are dependent on an intact G2/M checkpoint for survival, possibly due to inactivation of the G1/S checkpoint following a loss-of-function p53 mutation. Therefore, in such tumors, inhibition of WEE1 kinase in combination with DNA-damaging agents may result in mitotic catastrophe through accumulation of mutations and premature mitosis. A number of potent small-molecule WEE1 kinase inhibitors (WEE1i) have been identified through drug screening and used in preclinical and clinical trials [101]. The most developed of these is AZD1775, also known as adavosertib.

In the preclinical setting, AZD1775 has been shown to act synergistically with a range of chemotherapy agents in p53-deficient tumors and with ionizing radiation or cisplatin in medulloblastoma cells, irrespective of p53 phenotype [101]. WEEi may also work in conjunction with other DNA repair inhibitors; for example, the addition of WEE1i to ATRi therapy resensitized ATR-resistant cells through forced premature entry into mitosis [102]. Evidence for WEE1i monotherapy is weak however, and clinical trials predominantly evaluate WEE1i in combination with other therapeutic agents [101].

A phase I trial with AZD1775 in combination with various chemotherapy agents for patients with advanced solid tumors demonstrated encouraging efficacy, with higher response rates observed in p53-mutated patients [103]. A further phase II trial evaluated AZD1775 with carboplatin in the treatment of advanced platinum-resistant ovarian cancer; the ORR was 31.9% with a mPFS of 5.5 months. However, treatment-related toxicities, including gastrointestinal symptoms and cytopenias, were common and 12.8% of patients discontinued AZD1775 [104]. A phase II trial comparing AZD1775 in combination with gemcitabine to placebo showed significantly improved overall survival from 7.2 to 11.5 months (*P*=0.022), although hematological toxicity remained an issue [105]. Given the preclinical evidence suggesting WEE1i may be of most benefit in p53-mutated cancers, AZD1775 was assessed in combination with carboplatin and paclitaxcel for the treatment of p53-mutated, platinum-sensitive ovarian cancer. While there was no significant difference in response rates, mPFS was significantly greater in the AZD1775 arm as compared with placebo (9.9 vs 8.0 months; *P*=0.030) [106]. There is further promising evidence for triple combinations of AZD1775 with chemoradiotherapy regimens for both pancreatic and head and neck cancers [107].

Following concerns regarding the toxicity profile of WEE1i and chemotherapy agents, it has been suggested that WEE1i may be better tolerated in combination with precision anticancer therapies such as DNA repair inhibitors or immunotherapy. A phase Ib trial assessed the combination of AZD1775 and olaparib in 119 patients; despite demonstrating good efficacy, hematological toxicity was again common [108]. A randomized phase II trial in 273 metastatic TNBC patients found no significant differences in response rates or PFS with the addition of AZD1775 to olaparib alone (NCT03330847). A phase I trial found that the combination of AZD1775 with the PDL-1-inhibitor durvalumab, had an acceptable safety profile and evidence of antitumor activity [109]. A summary of clinical trials with WEE1i can be found in Table 3 [101–115]. Given these encouraging results and improved safety profiles, further trials of these novel combination therapies are warranted.

**Table 3:** Completed trials of WEE1 inhibitors

Study title	NCT trial number	Year completed	Cancer(s)	WEE1 inhibitor	Comparator	Sample size	Relevant results
A phase I trial of WEE1 inhibition with chemotherapy and radiotherapy as adjuvant treatment, and a window of opportunity trial with cisplatin in patients with head and neck cancer	NCT03028766	2021	Head and neck cancer	AZD1775 plus cisplatin plus radiotherapy	AZD1775 plus cisplatin	58	*Awaiting publication of results*
Phase Ib trial of dose-escalating AZD1775 in combination with concurrent radiation and cisplatin for intermediate and high risk head and neck squamous cell carcinoma (HNSCC) [108]	NCT02585973	2021	Head and neck cancer (SCC)	AZD1775 plus cisplatin plus radiotherapy	Nil	12	ORR: 100% at 3 months, mPFS and median overall survival were 90%
A phase Ib study combining irinotecan with AZD1775, a selective WEE1 inhibitor, in RAS (KRAS or NRAS) or BRAF mutated metastatic colorectal cancer patients who have progressed on first-line therapy	NCT02906059	2020	Colorectal	AZD1775 plus irinotecan	Nil	7	*Awaiting publication of results*
A phase II study of cisplatin + AZD1775 in metastatic triple-negative breast cancer and evaluation of pCDC2 as a biomarker of target response	NCT03012477	2020	Breast	AZD1775 plus cisplatin	Nil	34	ORR: 26% (95% CI: 13–44%). mPFS: 4.9 months (95% CI: 2.3–5.7)
A biomarker-enriched, randomized phase II trial of adavosertib (AZD1775) plus paclitaxel and carboplatin for women with platinum-sensitive TP53-mutant ovarian cancer [104]	NCT01357161	2020	Ovarian	AZD1775 plus paclitaxcel and carboplatin	Placebo plus paclitaxcel plus carboplatin	121	Adavosertib improved ePFS: 7.9 vs 7.3 months (*P*<0.2)
Open-label, multicenter, phase I study to assess safety and tolerability of adavosertib plus durvalumab in patients with advanced solid tumors [107]	NCT02617277	2019	Solid tumors	AZD1775 plus durvalumab	Nil	54	Disease control rate was 36%
Adavosertib with chemotherapy (CT) in patients (pts) with platinum-resistant ovarian cancer (PPROC): an open-label, four-arm, phase II study [102].	NCT02272790	2019	Ovarian	AZD1775 plus chemotherapy	Other chemotherapy regimens	94	ORR in combination with cisplatin was 67% with mPFS 10.1 months
A randomized double-blind placebo-controlled phase II trial comparing gemcitabine monotherapy to gemcitabine in combination with adavosertib in women with recurrent, platinum resistant epithelial ovarian cancer: a trial of the Princess Margaret, California, Chicago and Mayo Phase II Consortia [103].	NCT02151292	2019	Ovarian	AZD1775 plus gemcitabine	Gemcitabine alone	124	mPFS greater with AZD1775 (3.0–4.6 months, *P*=0.015) and overall survival (7.2–11.5 months, *P*=0.022). Greater response rate with AZD1775
Phase Ib study of adavosertib in combination with olaparib in patients with refractory solid tumors: dose escalation [106]	NCT02511795	2019	Solid tumors	AZD1775 plus olaparib	Nil	119	ORR: 11.1%; disease control rate: 55.7%
VIOLETTE: a randomized phase II study to assess the DNA damage response inhibitors AZD6738 or AZD1775 in combination with olaparib (Ola) versus Ola monotherapy in patients (pts) with metastatic, triple-negative breast cancer (TNBC).	NCT03330847	2019	Triple-negative breast cancer	AZD1775 plus olaparib	Olaparib alone	273	No significant difference in mPFS or ORR
A phase 2 study of WEE1 inhibition with AZD1775 alone or combined with cytarabine in patients with advanced acute myeloid leukemia and myelodysplastic syndrome	NCT02666950	2018	Acute myeloid leukemia and myelodysplastic syndrome	AZD1775	AZD1775 plus cytarabine	3	No responses seen
A phase I clinical trial of AZD1775 in combination with neoadjuvant weekly docetaxel and cisplatin prior to surgery in squamous cell carcinoma of the head and neck (HNSCC) [109]	NCT02508246	2018	Head and neck cancer (SCC)	AZD1775 plus cisplatin plus docetaxcel	Nil	10	Seven patients (70%) had a response. Two complete responses and four pathological responses
Dose escalation trial of the Wee1 inhibitor AZD1775, in combination with gemcitabine (+ radiation) for patients with unresectable adenocarcinoma of the pancreas [110]	NCT02037230	2018	Pancreatic	AZD1775 plus gemicibabine plus radiotherapy	Nil	34	Median overall survival: 21.7 months (90% CI: 16.7–24.8); mPFS: 9.4 months (90% CI: 8.0–9.9)
Phase II, single-arm study of AZD1775 monotherapy in relapsed small cell lung cancer patients [111]	NCT02593019	2018	Small-cell lung cancer	AZD1775	Nil	7	No objective responses, stable disease in three (42.9%)
A phase Ib, dose finding study evaluating AZD1775 in monotherapy, in combination with carboplatin and paclitaxel, and in combination with only carboplatin in adult asian patients with advanced solid tumours [112]	NCT02341456	2018	Solid tumors	AZD1775	AZD1775 plus cisplatin or paclitaxcel	19	Partial response in two patients (16.7%)
Phase I study evaluating WEE1 inhibitor AZD1775 as monotherapy and in combination with gemcitabine, cisplatin, or carboplatin in patients with advanced solid tumors [101]	NCT00648648	2016	Solid tumors	AZD1775	AZD1775 plus chemotherapy	173	Partial response in 17 patients (10%). Stable disease in 94 (53%)
A phase I study of single-agent AZD1775 (MK-1775), a Wee1 inhibitor, in patients with advanced refractory solid tumors [113]	NCT01748825	2015	Solid tumors	AZD1775	Nil	25	Two partial responses (8%) in BRCA cohort
A phase II study of AZD1775 plus pemetrexed and carboplatin followed by a randomised comparison of pemetrexed and carboplatin with or without AZD1775 in patients with previously untreated stage IV non-squamous non-small-cell lung cancer	NCT02087241	2015	Non-small-cell lung cancer	AZD1775 plus pemetrexed plus carboplatin	Placebo plus pemetrexed plus carboplatin	14 (terminated)	ORR 35.7% (5 responses from 14 patients before trial terminated)

Potential resistance mechanisms to WEE1i continue to be investigated, although suggested mechanisms include restoration of the G1/S cell-cycle checkpoint or up-regulation of other survival pathways in order to avoid mitotic catastrophe. One means of overcoming the former includes concurrent use of CDK4/6 inhibitors to remove the G1 checkpoint; this combination has been shown to act synergistically in the preclinical setting [116].

## Other preclinical synthetic lethality targets

### BER targets

As discussed above, BER plays an essential role in DNA repair. Up-regulation of BER is thought to contribute to chemoresistance, rationalizing the pathway as a pharmacological target. Further to this, the BER pathway may be a source of novel synthetic lethality targets as HR-deficient cells would lose the means of repairing both single- and double-strand breaks.

One emerging BER target is thought to be APE1; small-molecule inhibitors of the enzyme have been shown to be synthetically lethal *in vitro* to BRCA- and ATM-deficient cell lines [117]. APE1 is often overexpressed and associated with worse prognosis in NSCLC. In NSCLC cell lines, APE1 inhibition induced apoptosis, overcame chemotherapy resistance, and impeded cancer progression in a mouse model [118]. The APE1 inhibitor APX3330 was well tolerated in an early phase I trial, most commonly causing grade 1 fatigue, and demonstrated antitumor activity [119].

Similarly to APE1, FEN1 is often overexpressed in tumors and is particularly associated with development of chemoresistance. FEN1 inhibition was assessed in ovarian cancer cell lines and was demonstrated to potentiate cisplatin cytotoxicity as well as being synthetically lethal to BRCA2-deficient cells. In a similar manner to PARPi, resistance arose following restoration of BRCA2 function [120].

XRCC1 plays an integral role in the BER, SSBR, and back-up NHEJ pathways. Loss of XRCC1 has been shown to correlate with more aggressive cancers and worse prognosis. Intriguingly, PARP, ATM, ATR, WEE1, Mre11, and DNA-PKcs inhibitors have all been found to be synthetically lethal to XRCC1-deficient cells, highlighting a novel therapeutic avenue in these aggressive tumors [121–124].

### DNA polymerases

DNA polymerases, such as polβ and polθ, are integral components of DNA repair pathways. Polβ is vital for BER and hence maintaining genomic integrity. In ovarian cancer, high polβ expression was associated with worse patient outcomes while *in vitro* polβ depletion led to increased platinum sensitivity [125]. Furthermore, polβ inhibition has been shown to be synthetically lethal to both BRCA1- [126] and BRCA2-deficient cell lines [125]. Despite these promising findings, polβ inhibitors are yet to enter clinical trials possibly due to challenges in identifying suitably potent and specific inhibitors for *in vivo* use.

Polθ predominantly acts to repair DSBs through MMEJ, although recent evidence suggests that it may possess additional functions such as DNA cross-link repair or within the BER pathway. It is commonly overexpressed in many cancers, typically correlating with other HR defects and worse patient outcomes. Furthermore, overexpression of polθ has been shown to contribute to resistance to DNA-damaging agents such as radiotherapy, chemotherapy agents, and PARPi [31]. Following the discovery of a selective polθ inhibitor and using knockout models, it was identified that inhibition or loss of polθ can induce synthetic lethality in BRCA- and HR-deficient cells [31,127]. It has been suggested that polθ inhibitors may be of benefit in combination with other DNA repair inhibitors, such as PARPi and ATRi, as well as standard chemotherapy agents. For instance, loss of 53BP1 is thought to be a mechanism of PARPi resistance yet 53BP1 and polθ have been shown to be a synthetically lethal pairing. This therefore rationalizes combination strategies of PARP and polθ inhibitors as a means of preventing resistance. The polθ inhibitor ART4215 is the first to enter clinical trials and is being assessed for safety, tolerability, and preliminary efficacy in patients with advanced solid tumors, as both monotherapy and in combination with talazoparib or niraparib (NCT04991480).

### Other PARPi

The PARP family of proteins currently contains 17 members with wide-ranging cellular functions, including within DNA repair and mitosis. While currently licensed PARPi predominantly target PARP1 to PARP3, the other members of the PARP family may offer an avenue to novel therapies. The cellular functions and significance of each of these family members is reviewed in [128] but of particular clinical significance are PARP6 and PARP7. PARP6 inhibition has been shown to cause multipolar spindle (MPS) formation and centrosomal defects which in turn, caused cancer cell apoptosis both *in vitro* and *in vivo*. Additionally, PARP6 was identified to act on CHK1 and inhibition therefore prevents CHK1 modification, resulting in defective mitotic signaling [129]. PARP7 has a variety of roles but importantly loss-of-function results in increased microtubule stability leading to reduced mitotic rate as well as slowing of migration of ovarian cancer cells [128]. A selective PARP7 inhibitor, RBN-2397, has demonstrated good preclinical efficacy in lung cancer xenografts [130] and is now being evaluated in a phase I trial for treating advanced solid tumors (NCT04053673).

## Conclusion

DDR is a critical defense mechanism against genomic instability. Our current understanding of the DDR process has led to several translational investigations culminating in clinically viable precision oncology strategies. This is best exemplified by the current clinical use of PARPi in BRCA germline-deficient breast or ovarian cancers and platinum-sensitive sporadic epithelial ovarian cancers. However, response rate to PARPi is about 50% and progression-free survival is only about 7 months. Therefore, development of intrinsic and acquired resistance remains a clinical challenge. The development of biomarkers of response to PARP and other DDR inhibitor therapies remains an area of unmet clinical need. The importance and current development of validated, predictive biomarkers in relation to DDR inhibitors is reviewed in [131]. More recently to address these challenges, several new potential drugs such as those targeting ATM, ATR, WEE1, and others have emerged. These next-generation DNA repair inhibitors either as monotherapy or in combination with PAPR inhibitors could potentially improve outcomes but will need to be tested in phase III randomized trials in the future. Preclinically, several novel DNA repair targets are under evaluation. Finally, discovery of additional synthetic lethality interaction partners focused on DDR remains an area of intense investigation and will help advances in precision medicine strategies for cancer patients.

## Abbreviations

53BP1: p53-binding protein 1
AGT: O6-alkylguanine-DNA alkyltransferase
AP: apurinic/apyrimidinic
APE1: AP-endonuclease 1
ATM: ataxia telangiectasia mutated
ATMi: ATM inhibitor
ATR: ataxia telangiectasia and Rad3-related
ATRi: ATR inhibitor
ATRIP: ATR-interacting protein
BER: base excision repair
BRCT: BRCA1 C terminus
CHK1: checkpoint kinase 1
CPD: cyclobutane pyrimidine dimer
DDR: DNA damage signaling response and repair
DNA-PKcs: DNA-dependent protein kinase catalytic subunit
DSB: double-strand break
FA: fanconi anemia
FEN1: flap-endonuclease 1
GG-NER: global-genome NER
HNPCC: hereditary nonpolyposis colorectal cancer
HR: homologous recombination
ICL: interstrand cross-link
IDL: insertion/deletion loop
MGMT: methylguanine methyltransferase
MMEJ: microhomology-mediated end joining
MMR: mismatch repair
mPFS: medial progression-free survival
MPS: multipolar spindle
NER: nucleotide excision repair
PARP: poly(ADP-ribose) polymerase
PARPi: PARP inhibitor
PCNA: proliferating cell nuclear antigen
PDL-1: programmed death-ligand 1
PNKP: polynucleotide kinase/phosphatase
polβ: polymerase-β
PTEN: phosphate and tensin homolog
ROS: reactive oxygen species
RPA: replication protein A
SDSA: synthesis-dependent strand annealing
SSA: single-strand annealing
SSB: single-strand DNA break
SSBR: single-strand break repair
ssDNA: single-strand DNA
TC-NER: transcription-coupled NER
TNBC: triple-negative breast cancer
UV: ultraviolet
WEE1i: WEE1 kinase inhibitor
